# Associations Between Local Area Deprivation and Physical Activity Participation in People with Cognitive Impairment in the North East of England

**DOI:** 10.3233/JAD-230358

**Published:** 2023-08-29

**Authors:** Ríona Mc Ardle, Calum Hamilton, Silvia Del Din, Andrew Kingston, Louise Robinson, Brook Galna, Alan J. Thomas, Lynn Rochester

**Affiliations:** a Translational and Clinical Research Institute, Faculty of Medical Sciences, Newcastle University, Newcastle, UK; b Population Health Sciences Institute, Faculty of Medical Sciences, Newcastle University, Newcastle, UK; c Murdoch Applied Sports Science Laboratory, School of Allied Health, Murdoch University, Perth, Western Australia, Australia;2 Centre for Healthy Ageing, Health Futures Institute, Murdoch University, Perth, Western Australia, Australia; d Centre for Healthy Ageing, Health Futures Institute, Murdoch University, Perth, Western Australia, Australia

**Keywords:** Alzheimer’s disease, cognitive dysfunction, dementia, digital technology, exercise, home environment, walking, wearable electronic devices

## Abstract

**Background:**

Promoting physical activity, such as habitual walking behaviors, in people with cognitive impairment may support their ability to remain independent with a good quality of life for longer. However, people with cognitive impairment participate in less physical activity compared to cognitively unimpaired older adults. The local area in which people live may significantly impact abilities to participate in physical activity. For example, people who live in more deprived areas may have less safe and walkable routes.

**Objective:**

To examine this further, this study aimed to explore associations between local area deprivation and physical activity in people with cognitive impairment and cognitively unimpaired older adults (controls).

**Methods:**

87 participants with cognitive impairment (mild cognitive impairment or dementia) and 27 older adult controls from the North East of England were included in this analysis. Participants wore a tri-axial wearable accelerometer (AX3, Axivity) on their lower backs continuously for seven days. The primary physical activity outcome was daily step count. Individuals’ neighborhoods were linked to UK government area deprivation statistics. Hierarchical Bayesian models assessed the association between local area deprivation and daily step count in people with cognitive impairment and controls.

**Results:**

Key findings indicated that there was no association between local area deprivation and daily step count in people with cognitive impairment, but higher deprivation was associated with lower daily steps for controls.

**Conclusion:**

These findings suggest that cognitive impairment may be associated with lower participation in physical activity which supersedes the influence of local area deprivation observed in normal aging.

## INTRODUCTION

Approximately 50 million people are living with dementia worldwide, making the condition a global public health priority [1]. The World Health Organization recommend that people with dementia and mild cognitive impairment receive person-centered care and support to maintain their independence and quality of life for as long as possible [1]. Promoting physical activity may be an effective strategy to support these goals due to associations with improved quality of life and ability to carry out activities of daily living [2–5]. Physical activity does not have to include structured or planned activities such as exercise, but can be any movement produced by skeletal muscles requiring energy expenditure (e.g., walking in the home or community) [6]. As walking is the most common and accessible type of physical activity for under-served populations such as older adults [7, 8], research has focused on objectively capturing walking behaviors in people with cognitive impairment [9].

Evidence suggests that people with dementia participate in lower volumes of physical activity, such as taking fewer steps per day compared to normal ageing [9]. Additionally, people with dementia and mild cognitive impairment appear to have different patterns of physical activity compared to cognitively unimpaired older adults, spending proportionately more time in short walking bouts [10, 11]. Promotion of physical activity in people with cognitive impairment requires identification of factors which impact physical activity participation in this population. There is limited quantitative research examining this question, and most studies only consider disease-related predictors of physical activity such as cognitive and motor function [12]. However, van Alphen et al. [13]’s systematic review of qualitative literature proposed that the reasons people with cognitive impairment do or do not participate in physical activity are complex and multi-layered, including intrapersonal, interpersonal, environmental, and organizational factors. Notably, only a limited number of studies considered environmental factors [13].

The local area in which a person lives becomes an increasingly important influence on physical activity during aging, as reduced income, health, and mobility limit geographical movements [14]. People with cognitive impairment spend the majority of their walking time in short walking bouts (0–60 s) which are most likely take place in the home, while longer bouts (>60 s) may represent ambulation around the community and neighborhood [10, 15]. Complementary evidence reports that their movements outside of their home are severely restricted and mainly take place within the local vicinity [16]. Targeting physical activity in the local area may be more impactful than creating external opportunities (e.g., exercise classes). However, regular participation in physical activity is a health privilege, requiring time, money, security, and access [17]. Area deprivation may contribute to inequalities in physical activity participation through environmental factors; less deprived areas may have safer and walkable routes and more opportunities in the immediate community for social physical activity participation, therefore supporting physical activity [14]. There may also be associations between deprivation and activity within the home, reflecting effects beyond just the local environment (e.g., habit formation in earlier life, size and walkability of the home itself). Understanding this complex relationship may allow us to build more inclusive person-centered interventions and strategies to support people with cognitive impairment to remain physically active for longer, such as tailoring them to an individual’s personal characteristics and living circumstances.

To gain an initial understanding, this analysis aimed to explore associations between local area deprivation in the North East of England and the volume of physical activity in people with cognitive impairment and cognitively unimpaired older adults (controls). Our secondary aim is to explore associations between area deprivation and metrics relating to pattern of physical activity, as this may provide contextual information (e.g., as spending more time in longer bouts is likely to reflect time outside the home). We hypothesized that both people with cognitive impairment and controls living in more deprived areas would have a lower volume (i.e., fewer daily steps) and different patterns (i.e., shorter walking bouts) of ambulatory physical activity compared to those living in less deprived areas.

## METHODS

### Participants

This is a secondary analysis of the GaitDem study, which included 114 participants from the North East of England, including community-dwelling people with cognitive impairment (*n* = 87; including probable mild cognitive impairment and dementia due to Alzheimer’s disease, vascular dementia, dementia with Lewy bodies, and Parkinson’s disease dementia) and community-dwelling cognitively unimpaired older adults (controls; *n* = 27). Diagnosis of cognitive impairment and associated dementia disease conditions were verified by consensus clinical review; two clinicians independently reviewed participants’ clinical notes and study assessments to verify their clinical diagnosis. Where disagreements occurred, a third clinician reviewed participants’ notes and assessments to provide a consensus. Formal diagnostic criteria for Alzheimer’s disease [18], dementia with Lewy bodies [19], Parkinson’s disease dementia [20], and vascular dementia [21] were used to define and verify diagnosis of dementia. Diagnosis of mild cognitive impairment used standardized clinical or research criteria, with consideration of the underlying disease pathology such as Alzheimer’s disease [22], dementia with Lewy bodies [23], and Parkinson’s disease [24]. Eighteen (51%) of the Alzheimer’s disease group, eleven (38%) of those with dementia with Lewy bodies, seven (44%) of the Parkinson’s disease dementia, and three (43%) of the vascular dementia group had mild cognitive impairment at the time of assessment, based on a Clinical Dementia Rating of 0.5.

To be included in the study, participants had to be ≥60 years, and self-report the ability to walk for two minutes. Participants were excluded if they demonstrated any co-existing neurological conditions or movement disorders other than cognitive impairment and Parkinson’s disease, if they had drug-induced or vascular parkinsonism, severe mental illness, evidence of a stroke affecting motor function, or a poor command of English. Controls were cognitively unimpaired (Mini-Mental State Examination (MMSE) ≥25), functionally independent, without a diagnosis of cognitive impairment or Parkinson’s disease, and without medication or treatment for the aforementioned conditions.

Global cognition was measured using the standardized MMSE and Addenbrookes Cognitive Examination III (ACE-III). Other clinical and cognitive assessments have been previously reported [10, 25]. Ethics approval was granted by the NHS Local Research Ethics Committee, Newcastle and North Tyneside 1, Reference: 16/NE/005, IRAS project ID: 192941.

### Physical activity assessment

All participants were asked to wear a wearable sensor (Axivity AX3, York, UK) on their lower backs continuously for seven days. This analysis focused on three ambulatory physical activity characteristics across the domains of volume (i.e., daily step count) and pattern (i.e., mean bout length, alpha as described in Mc Ardle et al. [10] and Chastin et al. [26]). Alpha refers to the ratio of short to long walking bouts, scaled relative to an individual’s shortest walking bout. A high alpha score suggests that an individual’s total walking time is composed of proportionally shorter walking bouts compared to long. Data were extracted for each day of use. Data processing methods have previously been described in Mc Ardle et al. [10] and validated in Hickey et al. [27]. There was a minimum bout length of three consecutive steps applied, and any period of rest which was ≥2.5 s was considered resting time [28]. Additionally, physical activity data was aggregated into “short” bouts (<60 s) and “long bouts” (≥60 s). Any days without data were treated as missing but participants were retained for analysis given the data available for other days.

### Local area deprivation categorization

Individuals’ neighborhoods were linked to UK government English area deprivation statistics, stratified into fifths from 1 (neighborhood within the 20% least deprived areas of England) to 5 (neighborhood within the 20% most deprived areas of England), and cross-referenced to UK government rural/urban classifications. Local area deprivation is an overall relative measure of deprivation, constructed by combining seven domains of deprivation according to their respective weights, including 1) Income (22.5%), 2) Employment (22.5%), 3) education, skills, and training (13.5%), 4) health and disability (13.5%), 5) crime (9.3%), 6) barriers to housing and services (9.3%), and 7) living environment deprivation (9.3%).

### Data analysis

This is a secondary analysis of data derived from a study previously reported. The sample size represents those with data available from this previous work, and therefore there was no targeted recruitment from specific deprivation groups as indicated by any prior power analyses.

Mean and variance of daily physical activity data were jointly estimated with hierarchical log-linear (step count and bout length) or linear (alpha) models, adjusting for age and diagnostic group (cognitively impaired vs controls), with area deprivation level treated as an ordered category with four orthogonal polynomial terms, interacting with diagnostic group.

Zero-centered weakly informative normal priors were included for all regression coefficients, with further prior sensitivity analyses to confirm robustness of estimates. Models were checked for good convergence of sampling chains, with any sampling pathologies addressed as needed.

All analyses were undertaken with the *brms* package for *R* statistical software as an interface to the *Stan* probabilistic programming language [29].

## RESULTS

### Demographics

Less deprived areas were over-represented in this sample, with 38% of participants from the 20% least deprived areas versus 11% from the 20% most deprived areas: this was particularly evident for controls, for whom only one (4%) lived in a neighborhood within the 20% most deprived areas of England. Across the sample, most participants were drawn from urban, rather than rural areas.

### Associations between physical activity and local area deprivation

**Fig. 1:**
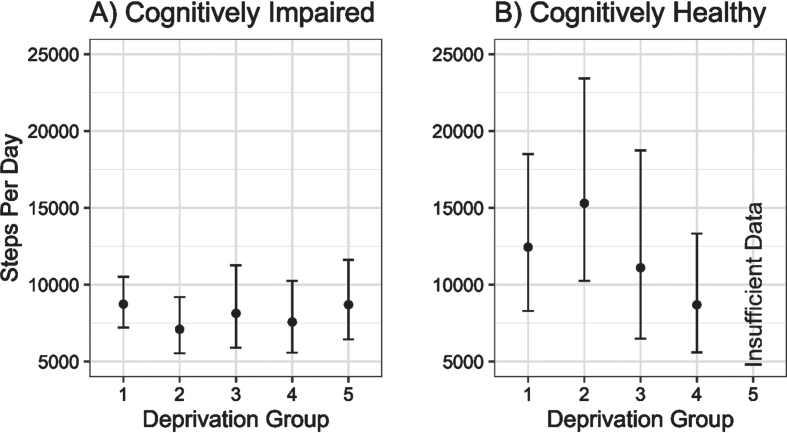
Estimated daily step counts (±95% CI) for cognitively impaired and cognitively unimpaired groups across deprivation fifths from 1 (least deprived) to 5 (most deprived).

There was little evidence of any association between increasing local area deprivation and changing physical activity volume (steps per day) in cognitively impaired older adults. In contrast, there was a non-linear association between increasing deprivation and physical activity volume in controls, reaching parity with the physical activity volume of the cognitively impaired group (see Fig. 1).

**Fig. 2:**
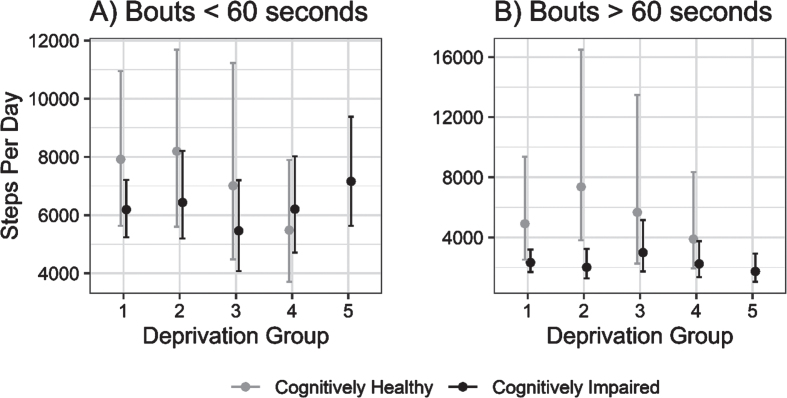
Estimated daily step counts (±95% CI) for cognitively impaired and cognitively unimpaired groups across deprivation fifths from 1 (least deprived) to 5 (most deprived) in ambulatory walking bouts under 60 s (A) and over 60 s (B).

Further exploratory analysis indicated that the differing role of local area deprivation on physical activity volume may be mediated by the length of ambulatory bouts; when isolating physical activity to short ambulatory bouts (i.e., <60 s), any deprivation-related differences were much less pronounced, though not entirely attenuated, in contrast to prolonged ambulatory bouts, as shown in Fig. 2.

**Fig. 3:**
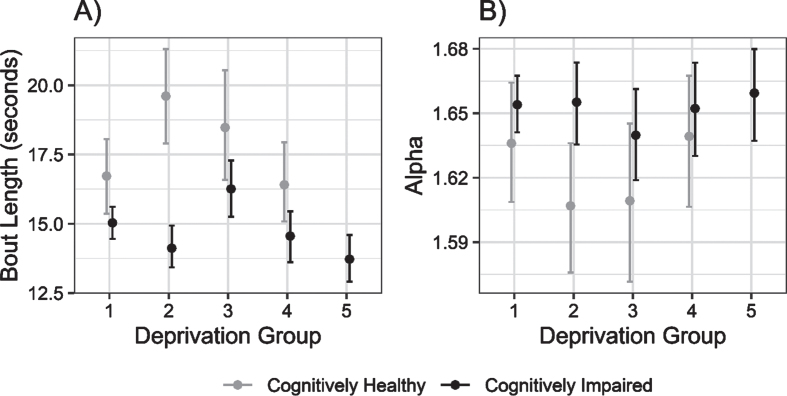
Mean (±95% CI) bout length and alpha (i.e., ratio of short to long walking bouts per individual) for cognitively unimpaired and cognitively impaired groups, across deprivation strata from least (1) to most (5) deprived areas.

Further exploratory analyses of metrics relating to the pattern of physical activity supported this link. Mean bout length was greater in controls than cognitively impaired people in less deprived areas, converging with increasing area deprivation. There was weak evidence of reduced mean bout length with increasing deprivation in cognitively impaired persons, although this was not consistent from group to group. Correspondingly, the alpha value was higher in the cognitively impaired group than controls in less deprived areas—indicating that they spent proportionately more time in shorter walking bouts—with the groups converging slightly with increasing area deprivation.

## DISCUSSION

This is the first study to investigate the role of local area deprivation on the volume and pattern of physical activity in cognitively impaired people compared to cognitively unimpaired older adults. Results indicate that cognitive impairment may be associated with lower participation in in ambulatory physical activity which supersedes the influence of local area deprivation observed in normal ageing. These findings were most pronounced in prolonged ambulatory bouts and may reflect reduced activity outside the home for people with cognitive impairment, consistent with GPS studies [30].

**Table 1:** Demographic table, categorized by local area deprivation fifths

	Indices of Multiple Deprivation Fifths				
	1 (Least Deprived),	2,	3,	4,	5 (Most Deprived),
	N = 43^1^	N = 23^1^	N = 16^1^	N = 19^1^	N = 13^1^
Age (y)	76	76	78	71	80
	(73, 82)	(72, 82)	(73, 81)	(68, 77)	(74, 84)
Diagnosis
Controls (*n* = 27)	8 (19%)	6 (26%)	5 (31%)	7 (37%)	1 (7.7%)
AD (*n* = 35)	16 (37%)	4 (17%)	5 (31%)	4 (21%)	6 (46%)
DLB (*n* = 29)	11 (26%)	7 (30%)	4 (25%)	6 (32%)	1 (7.7%)
PDD (*n* = 16)	6 (14%)	4 (17%)	1 (6.2%)	1 (5.3%)	4 (31%)
VaD (*n* = 7)	2 (4.7%)	2 (8.7%)	1 (6.2%)	1 (5.3%)	1 (7.7%)
Physical activity outcomes
Steps per Day	10,830 (4,843)	10,680 (4,917)	11,726 (6,873)	10,294 (4,751)	10,445 (4,129)
Mean Bout Length (s)	16.2 (3.8)	16.1 (4.7)	17.8 (3.7)	15.9 (3.4)	14.4 (2.6)
Alpha	1.650 (0.081)	1.650 (0.102)	1.632 (0.047)	1.648 (0.065)	1.653 (0.043)
Clinical outcomes
ACE-III Score/100	76	82	80	89	78
	(69, 88)	(71, 95)	(71, 88)	(72, 94)	(64, 83)
Missing	1	1	0	0	0
UPDRS-III Score	10	8	16	6	12
	(3, 28)	(3, 30)	(5, 27)	(2, 24)	(9, 28)
Missing	1	1	2	1	0
Body Mass Index (kg/m^2^)	24.8	25.9	26.3	27.4	27.7
	(23.8, 27.2)	(23.8, 28.5)	(23.1, 28.3)	(25.2, 32.8)	(24.2, 28.1)
Missing	0	0	0	1	0
Season of Assessment
Autumn	9 (21%)	4 (17%)	5 (31%)	1 (5.3%)	4 (31%)
Spring	8 (19%)	8 (35%)	7 (44%)	6 (32%)	4 (31%)
Summer	14 (33%)	8 (35%)	3 (19%)	7 (37%)	1 (7.7%)
Winter	12 (28%)	3 (13%)	1 (6.2%)	5 (26%)	4 (31%)
Urban Area	35 (81%)	20 (87%)	8 (50%)	14 (74%)	13 (100%)

^1^Mean (SD); Median (Lower Quartile, Upper Quartile); *n* (%). AD, Alzheimer’s disease; DLB, dementia with Lewy bodies; PDD, Parkinson’s disease dementia; VaD, vascular dementia; ACE-III, Addenbrookes Cognitive Examination-III; UPDRS-III, Unified Parkinson’s disease dementia rating scale-III.

Contrary to our hypothesis, people with cognitive impairment appeared to have similar daily step counts regardless of their local area deprivation level. In contrast, controls living in more deprived areas had lower volumes of physical activity compared to those living in less deprived areas. Differences in physical activity between the cognitively unimpaired and cognitively impaired groups in less deprived areas appeared to be driven by a greater step count in longer walking bouts (i.e., >60 s), which may reflect time spent out of the home [15, 28]. As research has indicated that people with cognitive impairment spend less time outside the home than normal ageing [30], this may account for the differences observed between our cognitively impaired and cognitively unimpaired groups. This raises the question of why people with cognitive impairment are spending less time outside of the home, which requires further socio-ecological consideration. Fear of falling and getting lost, loss of physical health, apathy, abilities to drive or independently use public transport, carers’ safety concerns, and requirement of carers to accompany people with cognitive impairment outside the home have all been cited as barriers to physical activity and may equally play a role in constraining individuals to their homes [13].

However, several assumptions were made about the data, such as longer walking bouts reflecting time spent outside of the home. Further research is required to validate these assumptions, such as capturing GPS data alongside physical activity measurement in this population. Supporting people with cognitive impairment to maintain their physical activity may be useful to decelerate decline in cognitive function, as a recent meta-analysis reported that physical activity moderates cognitive decline and reduces risk of declining to a more severely cognitively impaired state [31]. For consideration of future accessible, inclusive interventions, researchers should explore associations between independence, wellbeing, and health-related outcomes with physical activity both inside the home and outside the home. Given the associations between physical inactivity and increased falls risk, health and movement problems, and loss of social opportunities, it is vital that we support people with dementia to remain physically active and mobile for as long as possible [3, 5, 32]. Equally, the interaction between the environment a person lives and moves in with an individual’s intrapersonal and interpersonal experiences should be further explored, as promotion of physical activity requires socio-ecological considerations [13]. If people with cognitive impairment are primarily remaining within their own homes, home-based physical activity interventions may be feasible to implement, and may be effective at delaying cognitive decline and improving health and functional outcomes [33].

Notably, across the various physical activity measures, the effect of increasing local area deprivation in normal ageing was non-linear. This effect was attenuated in the least deprived group relative to levels 2 and 3. This could be explained as an artefact of the smaller sample sizes of the other deprivation groups (i.e., 1, 3, 5) leading to exaggerated estimates, or some true nonlinear effect explained by unseen factor in the least deprived areas (e.g., greater use of cars or taxis for transport rather than walking).

This study used a comprehensive approach to diagnosis of cognitive impairment by applying validated diagnostic criteria and a consensus approach by three clinicians. The use of validated algorithms to objectively measure physical activity also poses as a significant strength in this analysis. As this was a secondary data analysis, there are several limitations and potential avenues for further research. This study was limited by a small sample size and lack of regional diversity—only people living in the North East of England were included. Moreover, the dispersion of deprivation was limited, with only 4% of controls living in the most deprived areas compared to 14% of people with cognitive impairment. These exploratory results should therefore be interpreted cautiously and may provide indicators of the sample sizes required in future work with targeted recruitment from specific deprivation groups.

Additionally, the majority of participants lived in urban environments which have different walkability opportunities compared to rural areas, such as greater street connectivity and access to transport and services [34]; greater sampling of people living in rural and remote areas are required to understand the interaction between deprivation and urban/rural walking opportunities. Further work requires a more nationally representative sample which highlights the need for more inclusive recruitment strategies to insure a diverse study cohort. Additionally, this is an initial broad look at how physical activity might be influenced by one feature of an individual’s environment. Other features, such as access to recreational facilities, open green space, street connectivity, aesthetics, and safety have been associated with physical activity in older adults [35], and should be further explored in people with cognitive impairment. Finally, we did not consider the effect of dementia disease subtype or level of cognitive impairment on walking behaviors. Previous evidence has suggested that dementia subtypes with significant motor symptoms (e.g., Parkinson’s disease dementia) participate in lower volumes and demonstrate shorter and less variable walking bouts than Alzheimer’s disease [10], while patterns of walking activity appear affected by level of cognitive impairment [9]. As such, future research should consider the impact of local area deprivation on walking activity with consideration of dementia disease subtype and cognitive status, with an appropriately powered sample.

### Conclusions

This novel study demonstrated that contrary to normal ageing, volume of walking-based physical activity was not associated with local area deprivation in people with cognitive impairment from the North East of England. This may be due to people with cognitive impairment spending most of their time within their homes, with less frequent trips to their surrounding local area. Future work could explore this by collecting GPS data. This research should be replicated in a more diverse national sample including rural as well as urban settings. The promotion of physical activity within the home may be an accessible, inclusive intervention; further work is required to understand the clinical benefits this may have.

## FUNDING

This work is supported by the Alzheimer’s Society [ADSTC2014007] and the National Institute for Health Research (NIHR) Newcastle Biomedical Research Centre based at Newcastle upon Tyne Hospitals NHS Foundation Trust and Newcastle University [BH152398/PD0617]. Dr. Ríona Mc Ardle is funded by the National Institute for Health Research (NIHR) for her fellowship (NIHR 301677). The research was also supported by the National Institute for Health and Care Research (NIHR) Newcastle Biomedical Research Centre based at The Newcastle upon Tyne Hospitals NHS Foundation Trust, Newcastle University and the Cumbria, Northumberland and Tyne and Wear (CNTW) NHS Foundation Trust, and by the NIHR Research Capability Funding (NU-004071) for North East and North Cumbria. The views expressed are those of the authors and not necessarily those of the NHS, the NIHR, or the Department of Health.

## CONFLICT OF INTEREST

The authors have no conflict of interest to report.

## DATA AVAILABILITY

The data supporting the findings of this study are available on request from the corresponding author. The data are not publicly available due to privacy or ethical restrictions.
